# HA Triggers the Switch from MEK1 SUMOylation to Phosphorylation of the ERK Pathway in Influenza A Virus-Infected Cells and Facilitates Its Infection

**DOI:** 10.3389/fcimb.2017.00027

**Published:** 2017-02-07

**Authors:** Chengmin Wang, Huimin Liu, Jing Luo, Lin Chen, Meng Li, Wen Su, Na Zhao, Shelan Liu, Li Xie, Yaxiong Jia, Hua Ding, Xiufeng Wan, Hongxuan He

**Affiliations:** ^1^Key Lab of Animal Ecology and Conservation Biology and National Research Center for Wildlife-borne Diseases, Institute of Zoology, Chinese Academy of SciencesBeijing, China; ^2^Department of Infectious Diseases, Zhejiang Center for Disease Control and PreventionHangzhou, China; ^3^Department of Infectious Diseases, Hangzhou Center for Disease Control and PreventionHangzhou, China; ^4^Beijing Animal Husbandry StationBeijing, China; ^5^Institute for Genomics, Biocomputing and Biotechnology, Mississippi State UniversityMississippi, MS, USA

**Keywords:** MEK1, SUMOylation, influenza A virus, switch, hemagglutinin

## Abstract

Several post-translational modifications in host cells are hijacked by pathogens to facilitate their propagation. A number of components of the influenza virus have been reported to be modified by small ubiquitin-like modifier (SUMO) proteins during infection. We hypothesized that the MAPK/ERK pathway could be modified by SUMO1 because the SUMOylation of MEK1 was quickly eliminated after influenza A virus infection. We identified host cell MEK1 as a target of SUMO1 through LC/MS/MS, and enhanced MEK1 SUMOylation inhibited the infection of the virus, while inhibition of host cell MEK1 SUMOylation facilitated virus propagation. Further investigation demonstrated that the MAPK/ERK pathway is downregulated by MEK1 SUMOylation, which is inhibited by influenza virus infection. Furthermore, membrane accumulation of hemagglutinin promoted MEK1 phosphorylation and gradually abrogated the MEK1 SUMOylation. Taken together, we report a possible mechanism in which HA may trigger the ERK pathway in influenza A virus-infected cells as the switch from MEK1 SUMOylation to phosphorylation, facilitating virus infection.

## Introduction

Influenza A viruses can be highly contagious pathogens for both humans and several animal species. In support of their efficient replication within host cells, the viruses interact with a variety of host proteins and hijack cellular responses to antagonize several cellular pathways at different stages (Pleschka et al., 2001; Ludwig et al., 2006; Szretter et al., 2007). The post-translational conjugation of the Small Ubiquitin-like Modifier (SUMO) proteins to target proteins (SUMOylation) is an important mechanism for the regulation of protein activity, stability, cellular localization, and protein-protein interactions (Jürgen, 2004; Geiss-Friedlander and Melchior, 2007). Previous reports have indicated that influenza A virus interacts extensively with the cellular SUMOylation system during infection, and NS1, NS2, PB1, NP, and M1 proteins were targets of the cellular SUMOylation system (Pal et al., 2010, 2011; Xu et al., 2011). However, whether the host cellular SUMOylation status is affected by influenza A virus infection remains unexplored.

In eukaryotic cells, various extracellular stimuli generate intracellular signals that converge on a limited number of conserved protein kinase cascades, commonly referred to as the mitogen-activated protein kinase (MAPK) pathway. The MAPK cascade is recognized as a hallmark signaling pathway activated by influenza viruses (Pleschka et al., 2001), including the highly pathogenic avian influenza virus H5N1 (Droebner et al., 2011). The Raf/MEK/ERK signal cascade, which is activated during late influenza infection, is essential for efficient export of nuclear RNPs (Pleschka et al., 2001). Blockade of this pathway by specific inhibitors of MEK, or a dominant-negative growth of ERK or Raf resulted in a strongly impaired proliferation of influenza virus and retarded RNP export (Pleschka et al., 2001; Ludwig et al., 2006). Another study suggested that activation of the Raf/MEK/ERK cascade lead to efficient RNP export as well as virus production, achieved by the accumulation of the viral HA on the cell surface and enhanced viral polymerase activity (Marjuki et al., 2007). A detailed mechanism of the activation of the MAPK cascade and its relationship to SUMOylation remains elusive.

Because the host cellular SUMOylation system was hijacked by virus infection to modify numerous viral protein (Santos et al., 2013), the host cellular SUMOylation status might also be influenced by influenza virus infection. To better understand influenza virus-host interactions, we identified host cell MEK1 as a target of SUMO1, and the SUMOylation of MEK1 could be regulated by influenza A virus H5N1 infection. Enhanced MEK1 SUMOylation inhibited the infection of the virus, whereas downregulated-MEK1 SUMOylation facilitated virus propagation. Further investigation suggested that influenza virus infection impairs the MAPK/ERK (also called Ras-Raf-MEK-ERK) pathway by downregulating MEK1 SUMOylation. This study proposes a novel mechanism for influenza viruses to dampen the host response by abrogating the SUMOylation level of MEK1 to facilitate viral RNP export through the MAPK/ERK pathway.

## Materials and methods

### Viruses, cells, and plasmid constructs

Influenza virus A/environment/Qinghai/1/2008 (H5N1) was propagated in 9-day-old embryonated chicken eggs from specific-pathogen-free flocks (Beijing MERIAL Ltd.), at 37°C for 2 days. Allantoic fluid was clarified by centrifugation and stored at −80°C until use. Virus titer was determined by plaque assay. A549, MDCK, and HEK293T cells were routinely maintained in Dulbecco's Modified Eagle Medium (DMEM) supplemented with 10% fetal bovine serum, 100 μg/mL penicillin-streptomycin (HyClone, USA) and cultured at 37°C with 5% CO_2_. Cells were transiently transfected with Lip2000 transfection reagent (Invitrogen, USA). The dual expression plasmid constructs pcDNA5/FRT/TO/HA-MEK1/IRES/myc-Ubc9 (HA-MEK1-Ubc9), pcDNA3.1-Flag-SUMO1 (SUMO2/3, SUMO4), pcDNA3.1-Myc-Ubc9, and pcDNA3.1-HA-MEK1 were constructed by add-on PCR by insertion between *Bam*HI and *Xho*I sites of the pcDNA3.1(+) vector (Invitrogen). The MEK1^K64R^ mutant was generated by replacing lysine (K) 64 with arginine (R).

### HA-surface expression

MDCK cells were infected with different strains of influenza A virus as indicated (MOI = 1). Cells were incubated for 8 h. Then, the cells were detached with trypsin, fixed in PBS/4% paraformaldehyde, and a stepwise incubation with anti-H5-HA mAb and then with anti-mouse FITC-conjugated mAb (Calbiochem) each proceeded for 30 min on ice. Finally, HA-surface expression was determined by FACS analysis using an FACS Calibur (BD Biosciences).

### Immunoprecipitation and immunoblotting

A concentration of 2 × 10^6^ cells were lysed in 250 μL of ice-cold immunoprecipitation buffer (20 mM Tris-HCl, pH 8.0, 250 mM NaCl, 0.5% NP-40,3 mM EDTA, 0.1% SDS) freshly supplemented with 20 mM N-ethylmaleimide (NEM) and 5 mM iodoacetate (IAA) (Sigma, USA), 1 mM DTT (Sigma, USA) and complete protease inhibitor cocktail (Roche). Cell lysates (100 μg proteins) were then immunoprecipitated with indicated antibodies, and the proteins were separated with 7.5% SDS-PAGE for immunoblotting and visualized by a chemiluminescence reagent (Thermo, USA).

### Isolation and enrichment of SUMOylated proteins

The isolation and enrichment of SUMOylated proteins were performed with the SUMO-Qapture-T kit according to the manufacturer's instructions (Enzo, USA). Samples were prepared under native conditions in order to allow efficient binding of SUMO modified proteins to the SUMO-Qapture-T matrix. The eluted SUMO-protein conjugates were analyzed by LC-MS/MS (chip cube, Agilent Technologies, Waldbronn, Germany).

### SUMOylation assay

The *in vitro* SUMOylation assay kit was performed according to the manufacturer's instructions (BIOMOL, Goettingen, Germany). Briefly, reactions contained E1 enzyme (250 ng SAE1/SAE2), E2 enzyme (1.2 μg Ubc9), SUMO-1 or SUMO-2 protein (5.7 μg) and GST-fusion liker protein (5 μg) in the SUMOylation reaction buffer (55 mM Tris at pH 7.5, 5.5 mM MgCl_2_, 2.2 mM ATP, 5.5 mM DTT; dithiothreitol) and incubated at 37°C for 4 h. The reaction was terminated by adding 15 μl 2 × SDS-PAGE loading buffer and boiling at 95°C for 5 min. Samples were separated by probing with anti-SUMO1 antibody (Santa Cruz Biotechnology, Heidelberg, Germany) (1:1000) and detected by the ECL-plus detection system (GE Healthcare Bio-Sciences). Antibodies were washed out and the sample was reprobed with rabbit anti-SUMO-1 antibody (CST cell signaling technology).

### Cell treatment and immunoprecipitation of SUMOylated MEK1

Cells were treated with phorbol-12-myristate-13-acetate PMA (Sigma, USA). To detect SUMO-MEK *in vivo*, precleared cell lysates were rotated with anti-MEK1 antibodies at 4°C for 12 h. Immunoprecipitates, collected on protein A Sepharose, were washed 5 times with lysis buffer and subjected to SDS-PAGE and immunoblotting. For SUMOylated MEK1 detection in cells transiently transfected with HA-MEK1 and Flag-SUMO1, cell lysates were incubated with an anti-HA antibody (Cell Signalling Technology), precipitated with protein G Sepharose, washed 5 times with lysis buffer and analyzed by immunoblotting.

### *In situ* co-immunoprecipitation assay

HEK293 cells were transiently transfected with the appropriate plasmids and were lysed. Precleared cell lysates were incubated with an anti-Flag mAb (Cell Signalling Technology), then bound to protein G-Sepharose, and washed 4 times. Proteins were separated by SDS-PAGE, and immunoblotted with the indicated antibodies. To detect the endogenous MEK1-Ubc9 interaction, the precleared lysates were incubated with anti-MEK1 antibody at 4°C for 6 h. The immunoprecipitates were immunoblotted as described above.

### qRT-PCR quantification of NP gene specific mRNA

A549 cells were harvested and total RNA was extracted using Trizol reagent (GIBCO, USA). The RNA was reversely transcribed into cDNA using M-MLV reverse transcriptase (Promega, USA) and a poly-T oligonucleotide primer (5′-TTTTTTTTT TTTTTT-3′, Takara, Japan). The levels of NP mRNA transcripts were determined by quantitative real-time PCR using the SYBR Premix Ex Taq Kit (Takara). The PCR reactions (20 μL) were made in duplicate, performed at 95°C for 30 s, and subjected to 40 cycles of 95°C for 5 s and 60°C for 20 s on a Mx 3000P (Stratagene, USA). The sequences of the special primers were forward 5′-TTCATCAGAGGGACAAGA GTGG-3′ and reverse 5′-TCAGTTCAAGAGTGTTGG AGTC-3′ for NP (109 bp) and forward 5′-ATGTATCAGTTGTGGATCTGACCTG-3′ and reverse 5′-ATGCCTGCT TCACTACCTTCTTG-3′ for GAPDH (86 bp). The relative levels of NP mRNA transcripts for the control of GAPDH were analyzed with MxPro Q-PCR software and calculated by the double standard curve method.

## Results

### H5N1 virus infection down regulates MEK1 SUMOylation

**Figure 1:**
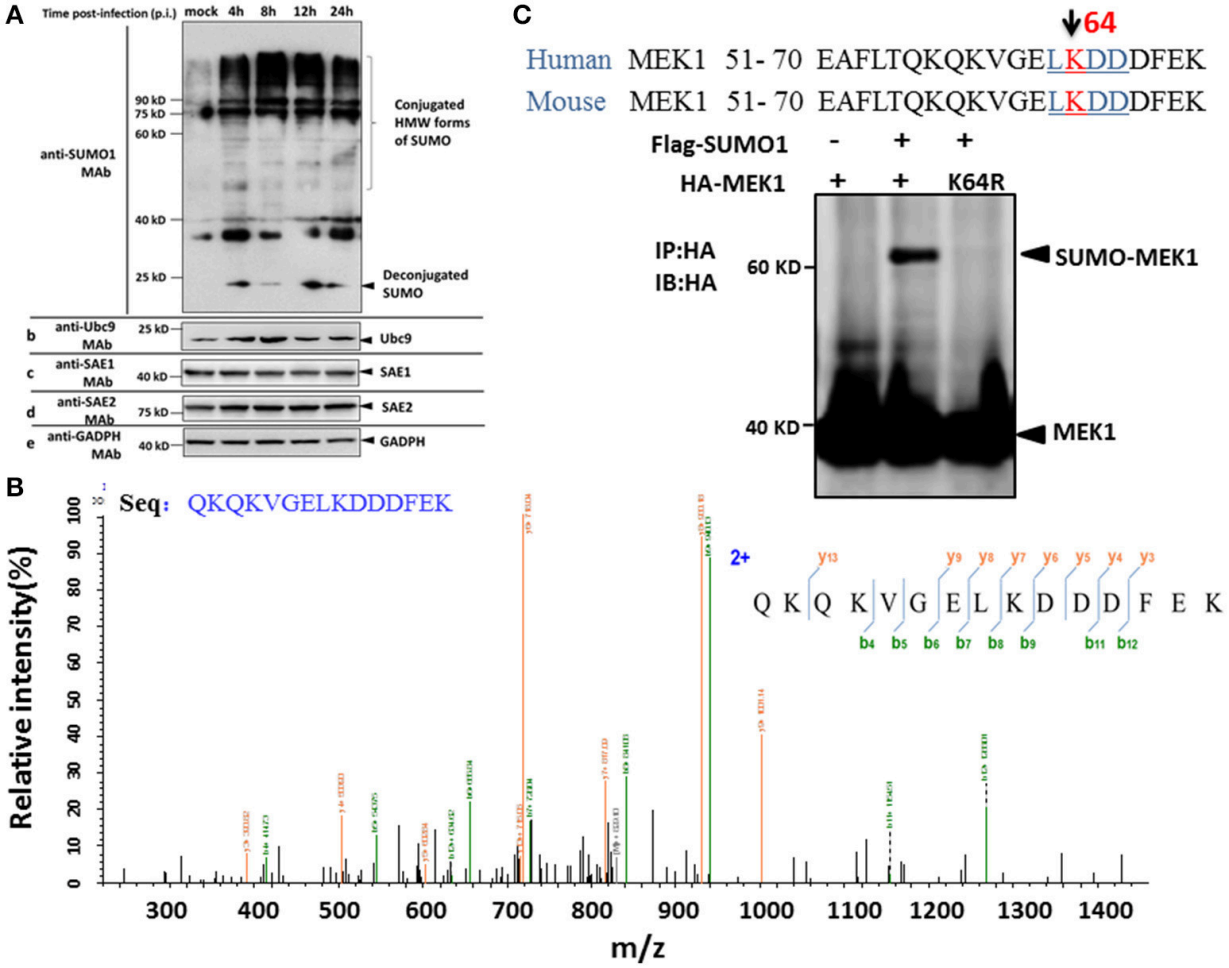
**SUMOylation of MEK1 is decreased in A549 cells after H5N1 influenza virus infection. (A)** A549 cells were infected with H5N1 at an MOI of 7, total cell extracts were collected at 4, 8, 12, and 24 h post infection, and analyzed by SDS-PAGE and western blotting using MAbs to SUMO1, Ubc9, SAE1, SAE2, and an internal control GAPDH. **(B)** Identification of the MEK1 SUMOylation site by LC-MS/MS analysis. **(C)** Alignment of MEK1 N-terminal amino acids from human and mouse. Bold and underlined text highlights the conserved LKDD motif.

To determine whether influenza virus infection affects the status of the host SUMOylation, A549 cells were infected with a high pathogenic virus strain [A/environment/Qinghai/1/2008(H5N1)] at a multiplicity of infection (MOI) of 7, and virus-infected cells were collected at 4, 8, 12, and 24 h post infection (p.i.) and subjected to immunoblotting analysis against SUMO1, Ubc9, SAE1, SAE2, and GADPH specific monoclonal antibodies. Compared to MOCK-infected cells, a predominant proportion of cellular SUMOylations increased substantially, but a small proportion of deconjugated SUMO increased at 4 h p.i. and then decreased slightly at 8 and 24 h post infection (Figure 1A). However, the cellular level of the SUMO conjugating enzyme Ubc9 did not demonstrate a corresponding increased (Figure 1A).

To identify differentially downregulated SUMOylated proteins involved in influenza virus infection, we used the A549 cell line to detect SUMO1 according to a previous report (Xu et al., 2011). Purification of differentially SUMOylated proteins coupled with LC-MS/MS identification was employed to investigate the potential changes of SUMOylated proteins in A549 cells in response to H5N1 virus infection (Figure 1B). Furthermore, we predicted the SUMO target by using the SUMOsp2.0 and SUMOplot Analysis Program. The results showed that K^64^ of MEK1 had the primary potential SUMO1 acceptation site 64, bearing the consensus motif of ψKxE, confirming MEK1 as a potential SUMO target (Figure 1C). Taken together, the host protein, MEK1 as a possible SUMO target, substantially decreased after H5N1 infection and the SUMOylation status of MEK1 might be important for influenza virus propagation.

### MEK1 interacts with Ubc9 and its SUMOylation is SUMO1 specific

**Figure 2:**
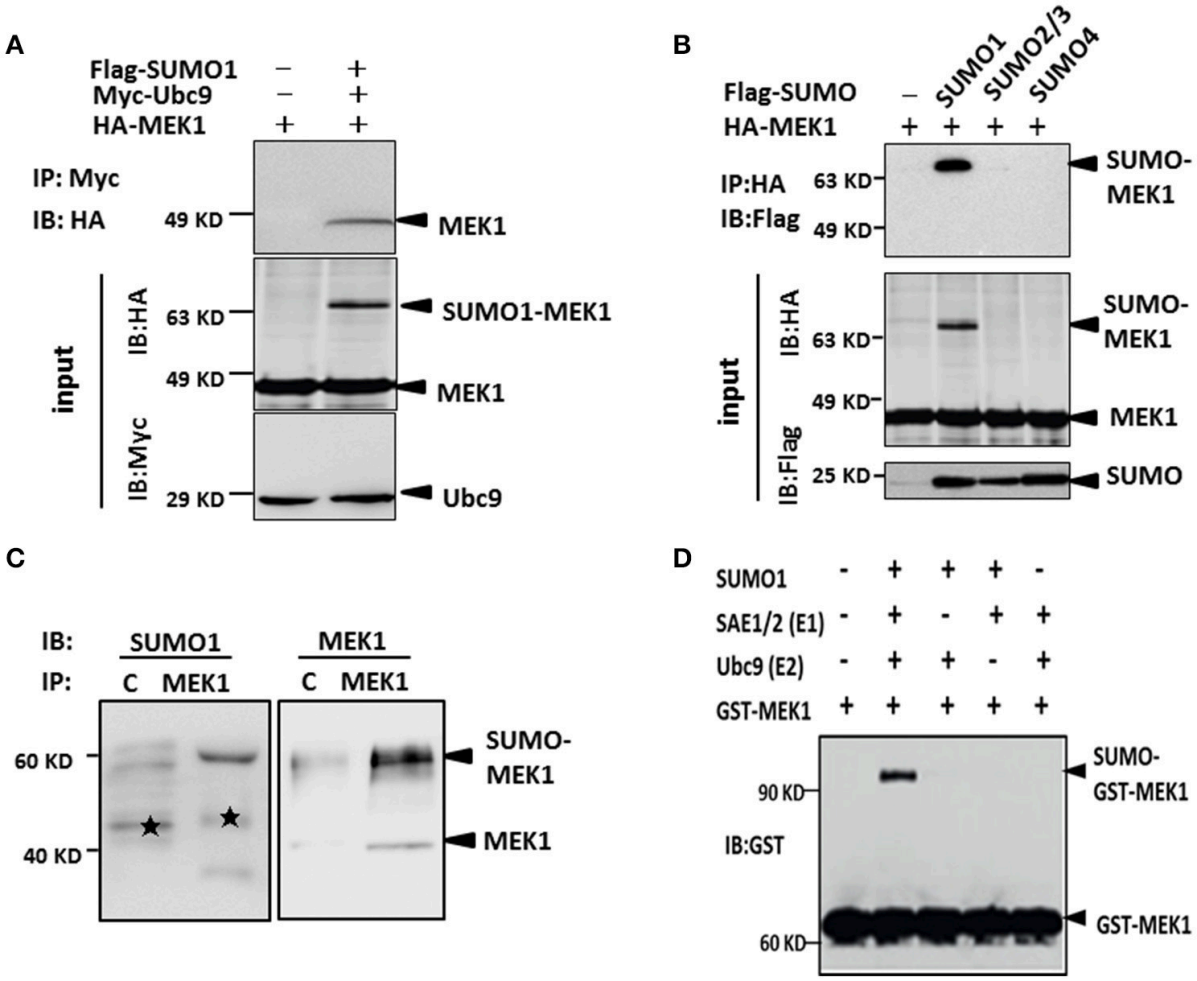
**MEK1 interacted with Ubc9 for SUMO1 conjugation. (A)** Cells lysates were immunoprecipitated with anti-c-Myc antibody and immunoblotted with anti-HA or Myc antibody. **(B)** HEK293 cells were co-transfected with HA-MEK1 and one of the Flag-tagged SUMO isoforms. Immunoprecipitated HA-MEK1 was either blotted with an anti-HA antibody or was probed for SUMOylation using an anti-Flag antibody. SUMO1 modification of endogenous MEK1 was observed in cells **(C,D)**.

As known, MEK1 as one of the two kinases, plays an important role in activation of the ERK-MAPK pathway (Kranenburg et al., 1999), which can be modified by influenza virus infection. SUMOylation sometimes requires direct interaction between Ubc9 and target proteins in order to transfer SUMO moiety from E1 to the substrates. To investigate whether MEK1 is in direct interaction with Ubc9, we co-transfected MEK1, Ubc9, and SUMO1 plasmids into HEK293 cells for 48 h, then immunoprecipitated Ubc9 by anti-Ubc9 antibody to finally determine whether MEK1 could be Co-IP or not. The results showed that MEK1-Ubc9 fusion protein can effectively utilize the cellular free SUMO1 protein, which lead to overexpression of MEK1SUMOylation through co-transfection and immunoprecipitation (Figure S1). We also found that MEK1 interacts directly with Ubc9 in the presence of SUMO1 (Figure 2A).

Furthermore, to determine whether the MEK1 undergoes SUMOylation *in vivo*, we expressed HA-MEK1 together with each one of the three Flag-tagged SUMO isoforms (SUMO1, SUMO2/3, and SUMO4) in HEK293 cells. HA-MEK1 was immunoprecipitated, separated by SDS-PAGE and immunoblotted with anti-HA or anti-Flag monoclonal antibodies (Figure 2B). A slower-migrating form of HA-MEK1/2, which was immunoreactive with the anti-Flag mAb, was detected only when co-transfected with SUMO1, indicating SUMO1 modification of MEK1. SUMO1 modification of endogenous MEK1 was observed in cells (Figures 2C,D). Taken together, these results suggest that MEK1 was specifically modified by SUMO1.

### SUMOylation of MEK1 downregulates Raf/MEK/ERK signaling

**Figure 3:**
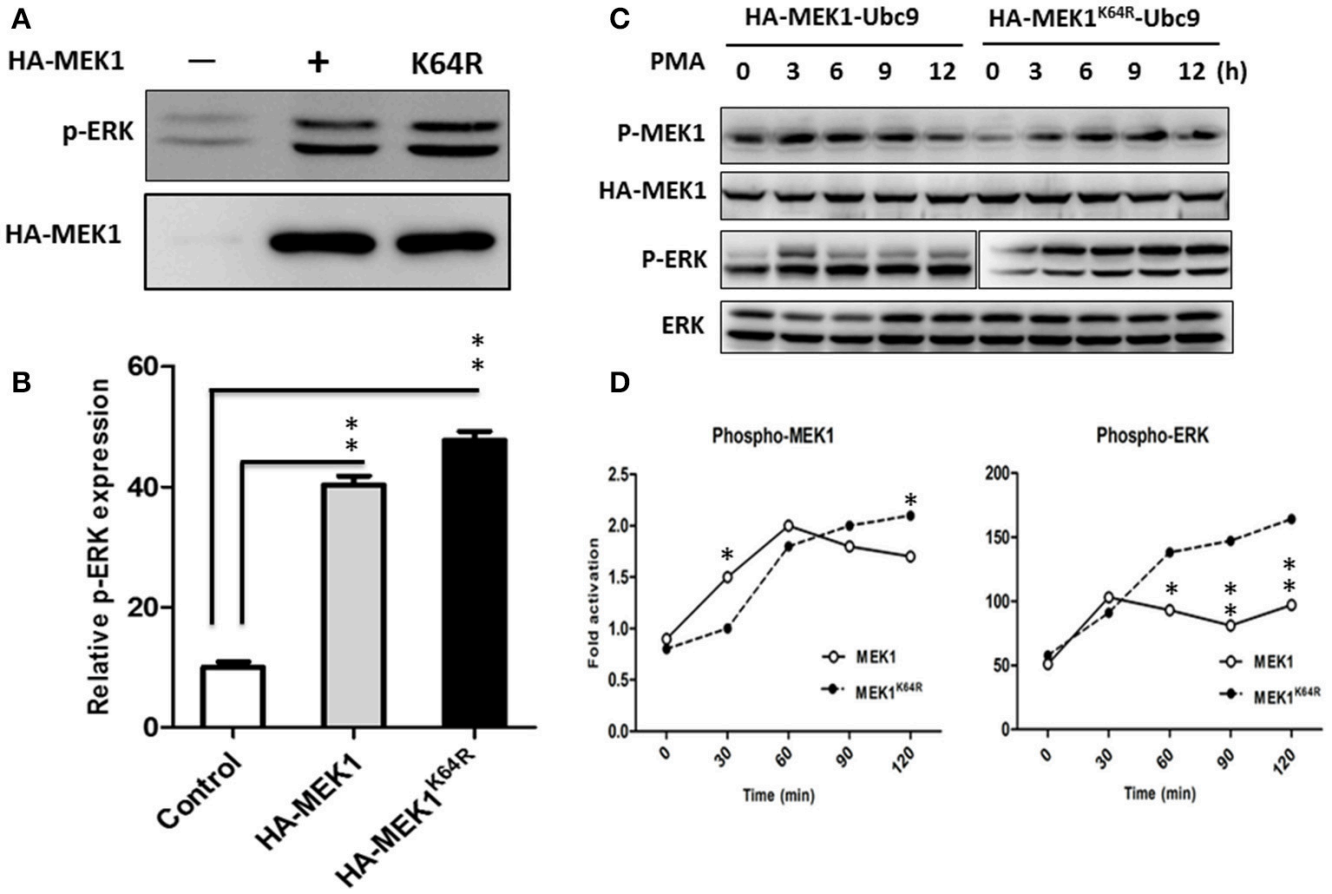
**HEK293 cells were transfected with HA-MEK1^K64R^ mutant and wild-type HA-MEK1. (A)** The expression levels of phospho-ERK and HA-MEK. **(B)** Phosphorylating activity of ERK was significantly higher in the mutant than in the wild type cell. **(C,D)** Cells that expressed HA-MEK1K^64^R elicited stronger ERK phosphorylation than cells that express wild type HA-MEK1. *P* values were calculated using a Student's *t*-test. ^*^P < 0.05; ^**^P < 0.01.

Although highly reversible and dynamic, a small proportion of SUMO conjugation results in a significant functional alteration of substrate protein through inter- or intra-molecular interactions (Geiss-Friedlander and Melchior, 2007). To determine whether MEK1 SUMOylation affects the Raf/MEK/ERK (MAPK) signal cascades, we compared the phosphorylating activity of ERK in wild-type cells with MEK1 and mutant cells with SUMOylation-defective MEK1^K64R^ which was generated by deactivating the SUMO binding site residue 64 in MEK1. Results showed that the phosphorylating activity of ERK was significantly higher in the mutant than that in wild-type cell (Figures 3A,B), indicating that SUMOylation significantly attenuates MEK activity in normally growing cells. In addition, we also found that MEK1 SUMOylation affects physiological processes for which the ERK pathway is crucial. Following phorbol-12-myristate-13-acetate (PMA) stimulation, HEK293 cells that express HA-MEK1^K64R^ elicited stronger ERK phosphorylation than cells that express wild type HA-MEK1 (Figures 3C,D), but overexpression of the MEK1-Ubc9 fusion protein did not exert a significant effect on MEK1 phosphorylation under the stimulation of PMA (Figure S2). Taken together, these data indicate MEK1 SUMOylation down regulates cellular functions mediated by ERK signaling.

### SUMOylation of MEK1 negatively controls the H5N1 virus propagation

**Figure 4:**
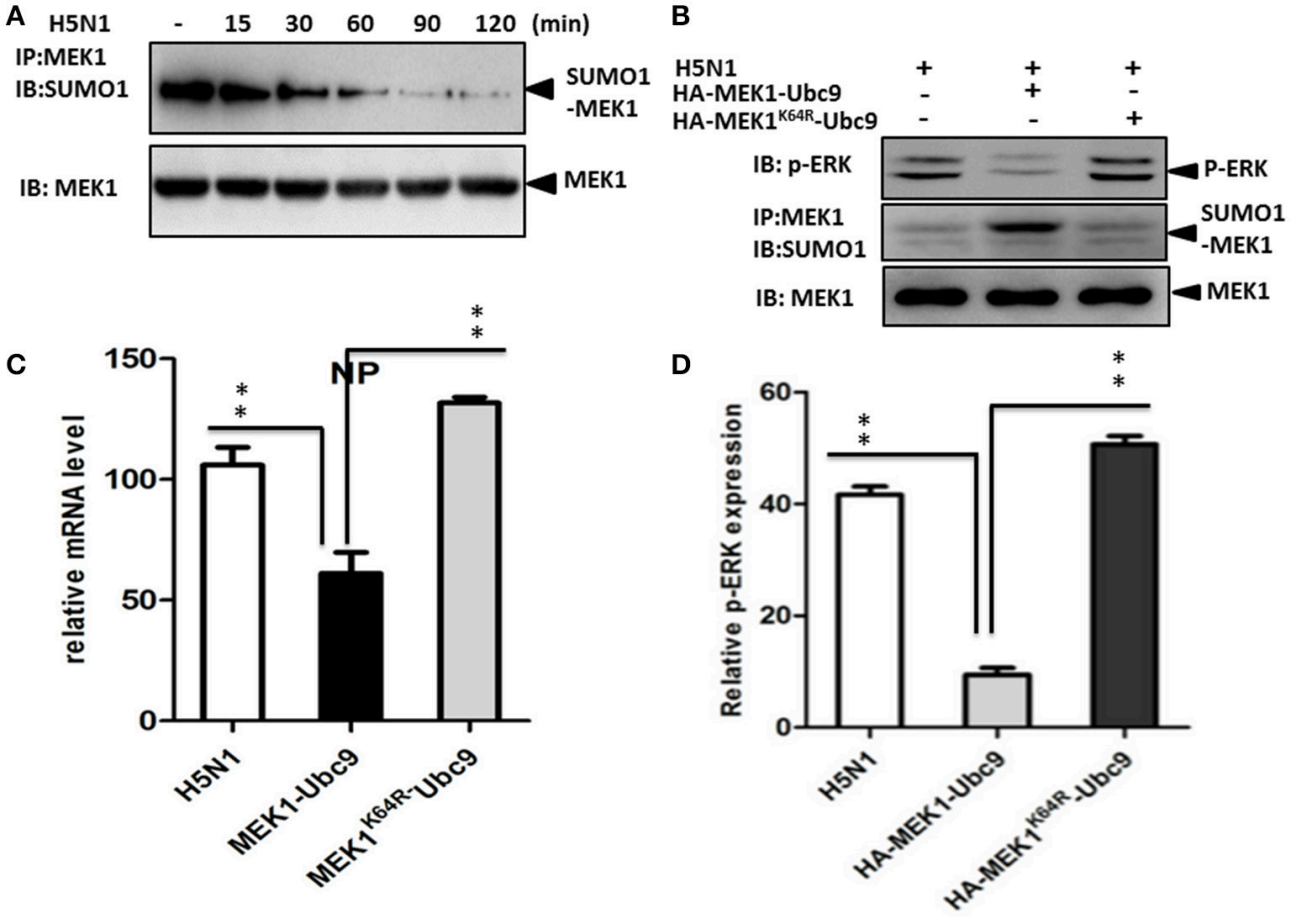
**SUMOylation of MEK1 negatively regulates virus propagation**. Cells infected by influenza virus showed almost completely inhibited MEK1 SUMOylation compared to MOCK cells without virus infection **(A)**. The endogenous ERK pathway was downregulated in HEK293 cells that stably expressed HA-MEK1-Ubc9 **(B)**. The NP gene expression level of influenza virus was downregulated significantly **(C)**. The endogenous ERK phosphorylation was downregulated by stable expression of HA-MEK1-Ubc9 in A549 cells after virus infection **(D)**. *P* values were calculated using a Student's *t*-test. ^*^P < 0.05; ^**^P < 0.01.

The influenza virus-induced Raf/MEK/ERK signal pathway is crucial for efficient virus replication, and blockade of this pathway reduces virus titers (Marjuki et al., 2007). To understand whether influenza virus infection can manipulate MEK1 SUMOylation, we measured endogenous MEK1 SUMOylation in virus-infected A549 cells. Surprisingly, MEK1 SUMOylation was almost completely abrogated in cells infected with influenza virus compared to in the MOCK cells without viral infection (Figure 4A). To examine whether overexpression of MEK1 SUMOylation would alter the outcome of virus infection, we constructed a dicistronic plasmid, HA-MEK1-Ubc9, which contains a sequence coding for an N-terminally HA-tagged MEK1 protein, the internal ribosome entry sites (IRES) from the encephalomyocarditis virus (EMCV), and N-terminally Myc-tagged Ubc9 protein (Figures 2A,C). We took advantage of the stable expression of HA-MEK1-Ubc9 in HEK293 cell to investigate whether overexpression of MEK1 SUMOylation can affect influenza virus propagation. After infection with an MOI of 7 [A/environment/Qinghai/1/2008(H5N1)], the endogenous ERK pathway was decreased in HEK293 cells expressed stably HA-MEK1-Ubc9 (Figure 4B). Simultaneously, the NP gene of the influenza virus was also decreased by 40.5%, which indicated that the virus propagation was significantly inhibited (Figure 4C). Stable expression of HA-MEK1-Ubc9 in A549 cells also downregulated endogenous ERK phosphorylation after H5N1 infection (Figure 4D). Additionally, we employed the stable expression of the HA-MEK1^K64R^-Ubc9 fusion protein in HEK293 cell to examine whether the mutation affects the H5N1 virus infection. After infection with an MOI of 10, HEK293 cells stably expressing HA-MEK1^K64R^-Ubc9 significantly upregulated the endogenous ERK pathway (Figure 4B) and the NP gene of H5N1 virus was increased by 110% indicating that the H5N1 propagation was enhanced (Figure 4C). Taken together, these data showed that MEK1 SUMOylation negatively regulates H5N1 propagation by ERK signaling.

### HA triggers the switch from MEK1 SUMOylation to phosphorylation in ERK pathway

**Figure 5:**
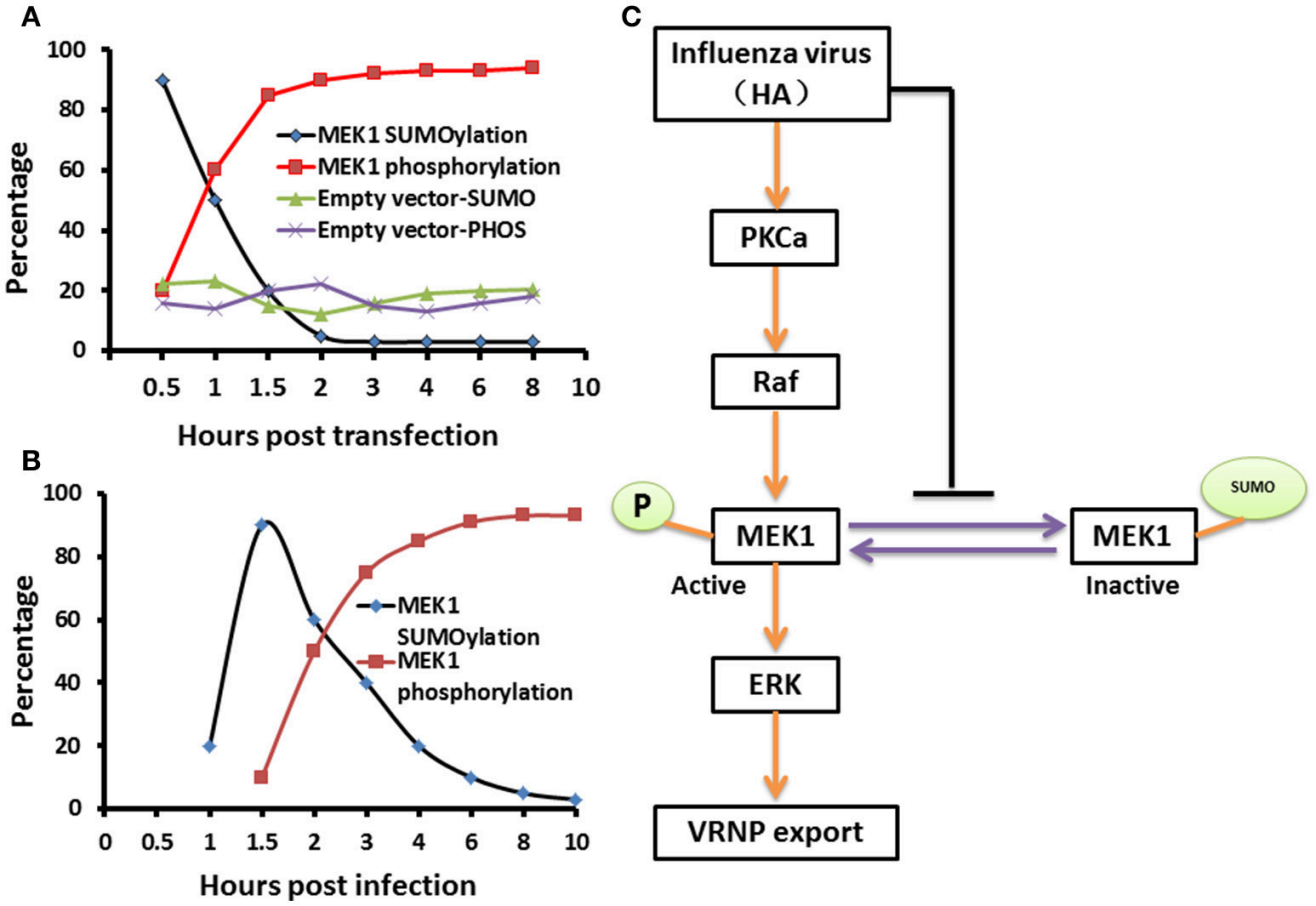
**A schematic model of negative regulation of the ERK pathway by MEK1 SUMOylation**. The dynamic changes of MEK1-SUMOylation and—phosphorylation show a similar trend for membrane expressing HA **(A)** and virus infection **(B)**, but the crossover point between treatments for virus infection appears 1 h later than when the membrane expresses HA. We propose a model that influenza virus activates the ERK pathway by negatively controlling MEK1 SUMOylation **(C)**. HA of influenza virus is essential for regulation of vRNP export in the ERK pathway through down regulation of MEK1 SUMOylation, namely, HA triggers the switch from MEK1 SUMOylation to phosphorylation of the ERK pathway and facilitates its infection.

The virus-induced MAPK signaling is activated late in the viral replication cycle, and RNP nuclear export is dependent on this cellular function (Pleschka et al., 2001; Ludwig et al., 2004). Membrane accumulation of influenza A virus hemagglutinin (HA) triggers nuclear export of the viral genome via protein kinase Cα-mediated activation of ERK signaling (Marjuki et al., 2007). To determine whether HA can play a role in the relationship between SUMOylation and phosphorylation of MEK1 in the ERK pathway, we constructed the expression vector pCAGGS-QH-HA expressing the QH wtH5-HA, and the MEK1-expression vector pKRSPA-HA-MEK1 (described previously, Ludwig et al., 1996, 1998) were used for transfection of MDCK cells. We found that the HA-membrane accumulation promoted the level of MEK1 phosphorylation and decreased the level of MEK1 SUMOylation at 1 h post infection, and the level of MEK1 SUMOylation reached a valley and the level of MEK1 phosphorylation reached a peak at 2 h post infection (Figure 5A and Figure S3). Furthermore, virus infection can also trigger a similar trend for MEK1 SUMOylation and phosphorylation (Figure 5B). Taken together, in normal cells, MEK1 SUMOylation attenuates the activity of MEK1 toward ERK, thereby inhibiting the ERK pathway. However, virus infection can induce the activation of ERK signaling by decreasing the MEK1 SUMOylation and increasing the MEK1 phosphorylation (Figure 5C).

## Discussion

Post-translational modifications provide a key functional regulatory link between the virus and host cell. Influenza virus infection can cause an increase of cellular SUMOylation, suggesting that SUMOylation plays an important role in virus-host interaction. The identification of SUMO targets affecting viral infection is fundamental to our understanding of virus-host interactions. Previous studies have demonstrated that the influenza viral proteins NS1, NS2, PB1, NP, and M1 are *bona fide* SUMO targets during viral infection (Pal et al., 2011). However, the mechanism underlying the interplay between host cellular SUMOylation proteins and influenza virus still remains unclear. In this study, we identified that MEK1, as a novel SUMOylation target protein after viral infection, physically interacts with Ubc9, and functions as a negative regulator in influenza virus-induced Raf/MEK/ERK signaling pathway. Our results show that MEK1 is modified specifically by SUMO1, and MEK1 SUMOylation down regulates the Raf/MEK/ERK pathway. In addition, we provided evidence to support that MEK1 functions in concert with Ubc9 to suppress ERK signaling, thereby negatively controlling influenza virus. Importantly, our findings suggest that MEK1 SUMOylation represses Raf/MEK/ERK signaling pathway, thereby downregulating influenza virus multiplication. Thus, our study not only uncovers a novel mechanism by which influenza virus hijacks the cellular SUMOylation pathway for its propagation but also provides novel insights into the link between host cellular SUMOylation and influenza virus infection (Figure 5).

SUMO modification is a highly reversible and dynamic process, the SUMOylation status of several target proteins changes markedly in response to various stimuli and/or depending on their subcellular localization (Dadke et al., 2007). We noted that the mutant form of MEK1, MEK1^K64R^, is resistant to MEK1 SUMOylation, thereby activating the ERK signaling pathway. Indeed, the MEK1^K64R^ mutant was more potent than their wild-type MEK1 in activating ERK *in vivo*. Therefore, inhibition of MEK1 activity by SUMOylation, in concert with protein phosphatase-mediated inhibition, may control the magnitude and duration of ERK activity. The regulation is biologically important because the magnitude and duration of ERK activity are critical determinants of cell fate, such as differentiation, growth and survival.

Influenza viruses exploit host cell SUMOylation machinery for their own propagation (Marusic et al., 2010; Wu et al., 2011; Xu et al., 2011). Small hepatitis delta antigens (S-HDAg) are modified by the SUMO-1 protein and then SUMOylated S-HDAg selectively enhances HDV genomic RNA and mRNA synthesis but not antigenomic RNA synthesis (Tseng et al., 2010). Viroplasm proteins of rotavirus are also SUMOylated to modulate virus replication (Campagna et al., 2013). Epstein–Barr virus utilizes the cellular SUMOylation system for its own benefit (Murata et al., 2010; Callegari et al., 2014). SUMOylation of nonstructural 5A protein regulates hepatitis C virus replication by cellular SUMO modification system in both HEK293T cells and Huh7.5 cells (Lee et al., 2014). Adult T-cell leukemia/lymphoma (ATL) response to arsenic/interferon therapy is triggered by PML-dependent hyper-SUMOylation by small ubiquitin-like modifier (SUMO)2/3, ubiquitination by RNF4, and proteasome-dependent degradation (Dassouki et al., 2015). Therefore, SUMOylation is one of the post-translational modifications involved in many cellular processes, including transcription activity, protein stability, nuclear-cytosolic localization and protein–protein interaction (Meulmeester and Melchior, 2008).

Influenza A virus is responsible for millions of infections and thousands of deaths in the human population worldwide each year. It extensively exploits and manipulates host cell functions to support viral replication, and therefore, uncovering these dependencies could help to develop new strategies for antiviral therapy. We showed that influenza virus H5N1-induced activation of the Raf/MEK/ERK cascades inhibited MEK SUMOylation to enhance viral replication. Given the well-defined role of the ERK pathway in influenza virus infection, therapeutic targeting of ERK signaling components is an area of intense investigation. It may be feasible to specifically block ERK signaling by augmentation of MEK SUMOylation, thereby providing a novel strategy for antiviral control of the virus.

## Author contributions

CW, HH, and HL conceived the study, and HL performed the experiments described in Figure 1. CW performed and JL and LC directed the experiments described in Figure 2. ML, NZ, and WS performed experiments described in Figure 3. SL, LX, and YJ performed experiments described in Figure 4. HD and CW designed pathway described in Figure 5. HL, CW, JL, and HH wrote the manuscript and XW, HH edit and revise the manuscript.

### Conflict of interest statement

The authors declare that the research was conducted in the absence of any commercial or financial relationships that could be construed as a potential conflict of interest.

## Supplementary material

Figure S1 **Dicistronic expression constructs HA-MEK1-Ubc9 used in this study**. MEK1-Ubc9 fusion protein can effectively utilize free intracellular SUMO1 molecules and cause overexpression of MEK1 SUMOylation **(A)**; the immunoprecipitation results showed that the co-transfection expression vector of MEK1-Ubc9 can be combined with intracellular SUMO1 molecules **(B)**.

Figure S2 **MEK1 SUMOylation can be phosphorylated *in vitro***. HA-MEK1-Ubc9 was immunoprecipitated from stably transfected HEK293 cells that were treated with PMA (10 μg/ml) for the indicated times and were probed with phospho-MEK1 (P-MEK1) or anti-HA (HA) antibodies **(A)**. The intensity of the P-MEK1bands was quantified **(B)**.

Figure S3 **Analysis of HA-Surface Expression**. A549 cells were infected with influenza A virus as indicated (m.o.i. = 1). Cells were incubated for 8 h. Then, the cells were detached with trypsin, fixed in PBS/4% paraformaldehyde, and stepwise incubated with anti-H5-HA mAb and then with anti-mouse FITC-conjugated mAb (Calbiochem) for 30 min on ice each. Finally, HA-surface expression was determined by FACS analysis using an FACS Calibur at 3, 6, 9, and 12 h.
