# Supplementary figures and images for: HA Triggers the Switch from MEK1 SUMOylation to Phosphorylation of the ERK Pathway in Influenza A Virus-Infected Cells and Facilitates Its Infection
Source: Front Cell Infect Microbiol. 2017 Feb 7;7:27. doi: 10.3389/fcimb.2017.00027 (PMC5293797; doi:10.3389/fcimb.2017.00027)

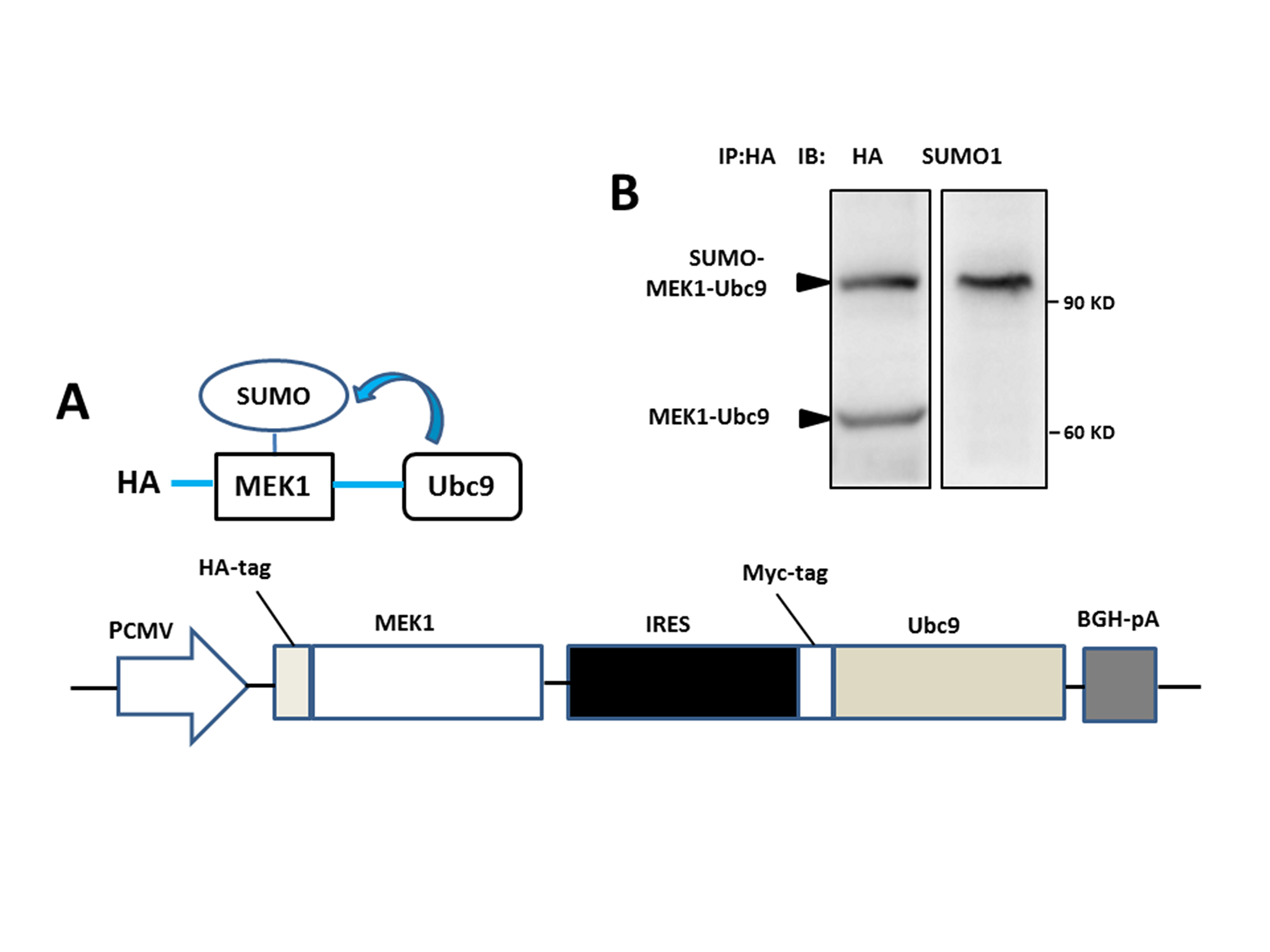

Supplement: Figure S1 — Dicistronic expression constructs HA-MEK1-Ubc9 used in this study. MEK1-Ubc9 fusion protein can effectively utilize free intracellular SUMO1 molecules and cause overexpression of MEK1 SUMOylation (A); the immunoprecipitation results showed that the co-transfection expression vector of MEK1-Ubc9 can be combined with intracellular SUMO1 molecules (B). [file Image1.TIF]

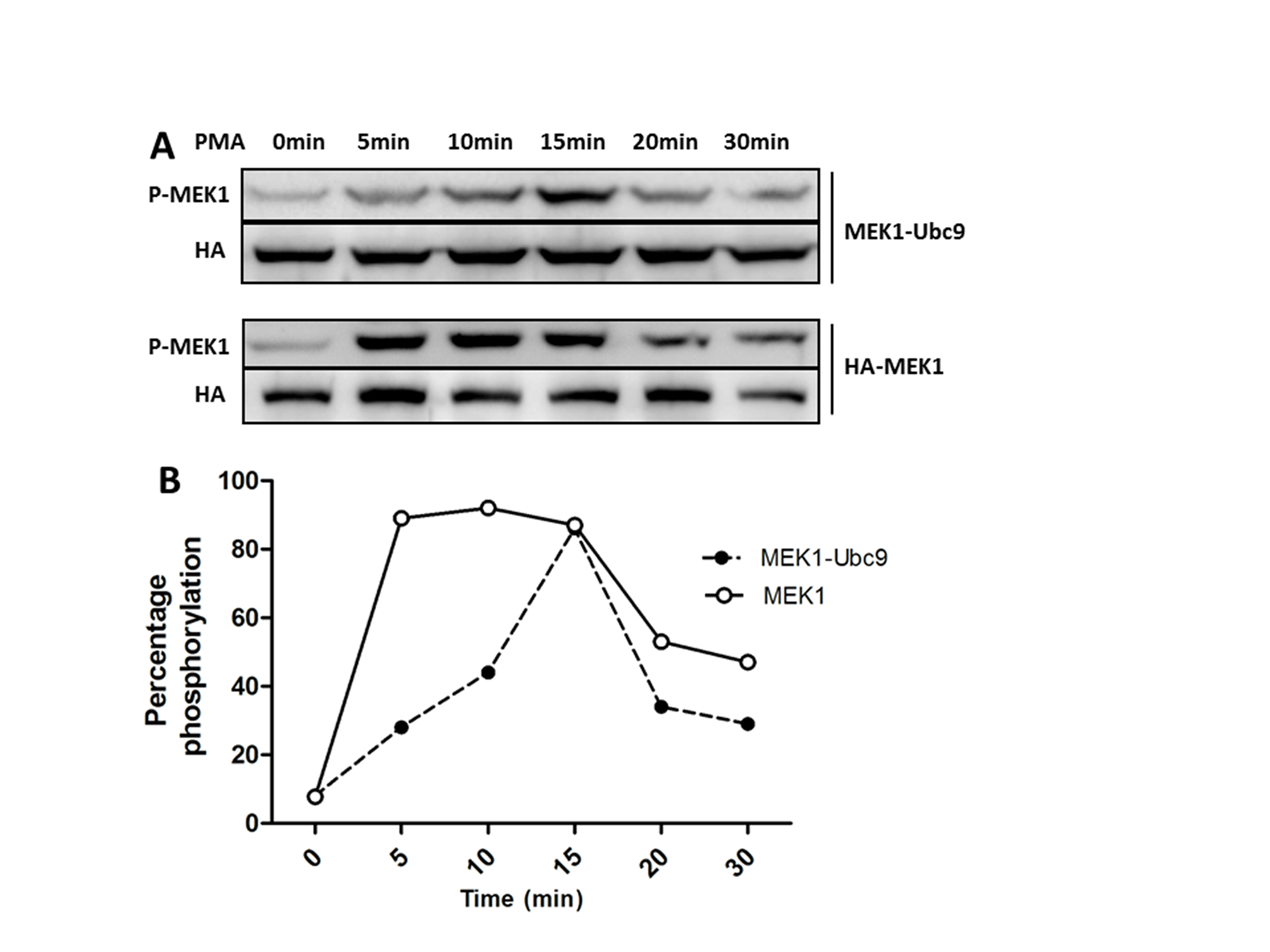

Supplement: Figure S2 — MEK1 SUMOylation can be phosphorylated in vitro. HA-MEK1-Ubc9 was immunoprecipitated from stably transfected HEK293 cells that were treated with PMA (10 μg/ml) for the indicated times and were probed with phospho-MEK1 (P-MEK1) or anti-HA (HA) antibodies (A). The intensity of the P-MEK1bands was quantified (B). [file Image2.TIF]

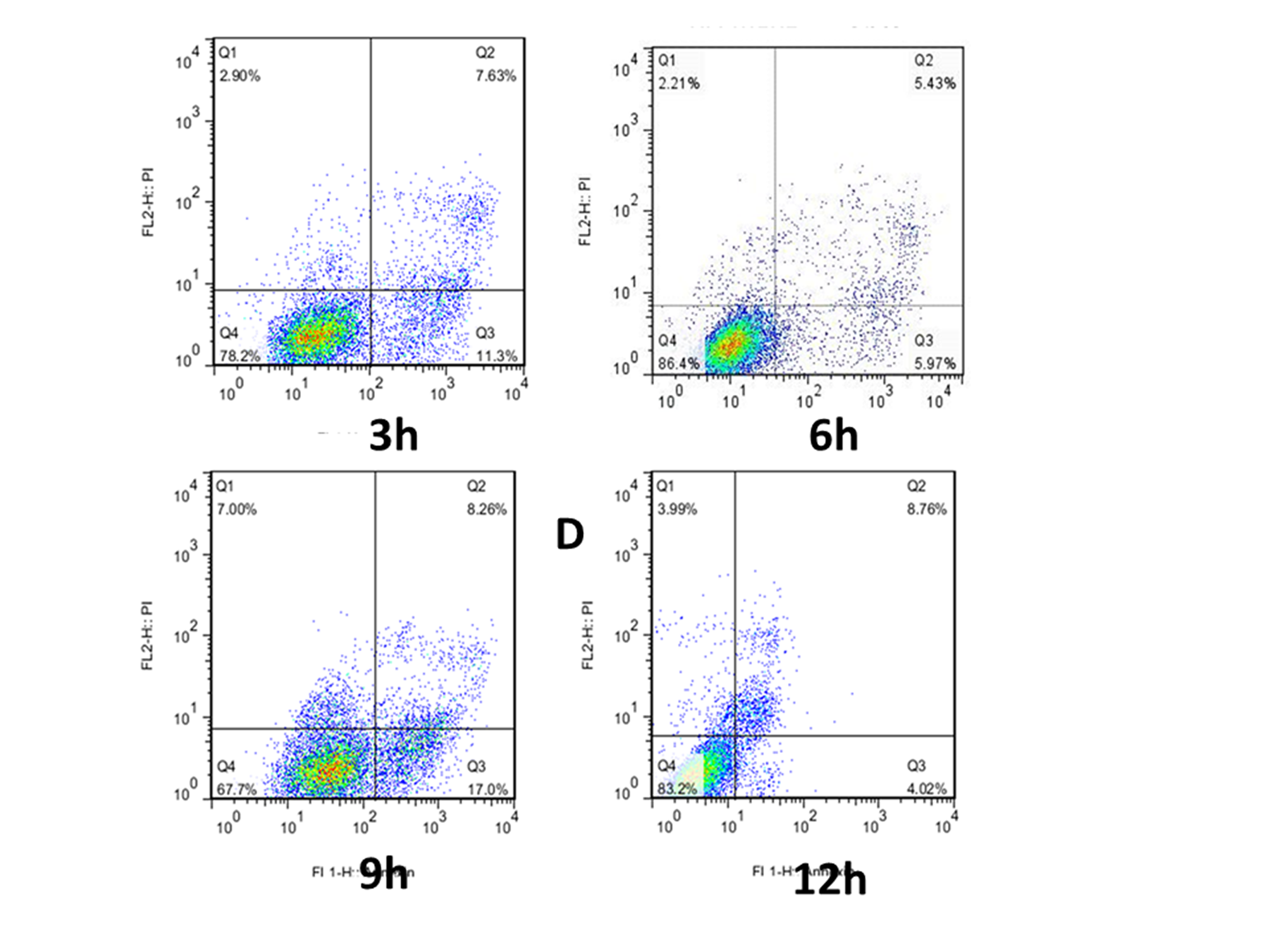

Supplement: Figure S3 — Analysis of HA-Surface Expression. A549 cells were infected with influenza A virus as indicated (m.o.i. = 1). Cells were incubated for 8 h. Then, the cells were detached with trypsin, fixed in PBS/4% paraformaldehyde, and stepwise incubated with anti-H5-HA mAb and then with anti-mouse FITC-conjugated mAb (Calbiochem) for 30 min on ice each. Finally, HA-surface expression was determined by FACS analysis using an FACS Calibur at 3, 6, 9, and 12 h. [file Image3.TIF]
